# Distribution and Numbers of Pygmies in Central African Forests

**DOI:** 10.1371/journal.pone.0144499

**Published:** 2016-01-06

**Authors:** Jesús Olivero, Julia E. Fa, Miguel A. Farfán, Jerome Lewis, Barry Hewlett, Thomas Breuer, Giuseppe M. Carpaneto, María Fernández, Francesco Germi, Shiho Hattori, Josephine Head, Mitsuo Ichikawa, Koichi Kitanaishi, Jessica Knights, Naoki Matsuura, Andrea Migliano, Barbara Nese, Andrew Noss, Dieudonné Ongbwa Ekoumou, Pascale Paulin, Raimundo Real, Mike Riddell, Edward G. J. Stevenson, Mikako Toda, J. Mario Vargas, Hirokazu Yasuoka, Robert Nasi

**Affiliations:** 1 Grupo de Biogeografía, Diversidad y Conservación, Departamento de Biología Animal, Universidad de Málaga, Facultad de Ciencias, Málaga, Spain; 2 Division of Biology and Conservation Ecology, School of Science and the Environment, Manchester Metropolitan University, Manchester, United Kingdom; 3 Center for International Forestry Research (CIFOR), CIFOR Headquarters, Bogor, Indonesia; 4 Department of Anthropology, University College London, London, United Kingdom; 5 Department of Anthropology, Washington State University, Vancouver, Washington, United States of America; 6 Global Conservation Program, Wildlife Conservation Society, Bronx, New York, United States of America; 7 Dipartimento di Scienze, Università Roma Tre, Rome, Italy; 8 Asociación Zerca y Lejos, Madrid, Spain; 9 46 Elm Row, Edinburgh, United Kingdom; 10 Faculty of International Studies, Tenri University, Tenri City, Nara, Japan; 11 Chameleon Strategy, London, United Kingdom; 12 46 Yoshida-Shimoadachi, Sakyo, Kyoto, Japan; 13 Faculty of Education, Yamaguchi University, Yoshida, Yamaguchi-shi Yamaguchi, Japan; 14 Graduate School of Asian and African Area Studies, Kyoto University, Shimoadachi-cho, Yoshida, Sakyo-ku, Kyoto, Japan; 15 COOPI-Cooperazione Internazionale ONG Onlus, Milano–I, Italy; 16 Department of Geography, University of Florida, Gainesville, Florida, United States of America; 17 Institut Santé et Société, Université du Québec à Montréal, Montréal, Québec, Canada; 18 DDL Lab. CNRS—Université Lumière Lyon 2, Lyon, France; 19 Bioclimate, Research and Development, Edinburgh, United Kingdom; 20 Department of Anthropology, Durham University, Durham, United Kingdom; 21 Faculty of Humanity and Environment, Hosei University, Fujimi, Chiyoda-ku, Tokyo, Japan; 22 Consultative Group on International Agricultural Research (CGIAR), CIFOR Headquarters, Jalan CIFOR, Situ Gede, Bogor, Indonesia; University of Florence, ITALY

## Abstract

Pygmy populations occupy a vast territory extending west-to-east along the central African belt from the Congo Basin to Lake Victoria. However, their numbers and actual distribution is not known precisely. Here, we undertake this task by using locational data and population sizes for an unprecedented number of known Pygmy camps and settlements (*n* = 654) in five of the nine countries where currently distributed. With these data we develop spatial distribution models based on the favourability function, which distinguish areas with favourable environmental conditions from those less suitable for Pygmy presence. Highly favourable areas were significantly explained by presence of tropical forests, and by lower human pressure variables. For documented Pygmy settlements, we use the relationship between observed population sizes and predicted favourability values to estimate the total Pygmy population throughout Central Africa. We estimate that around 920,000 Pygmies (over 60% in DRC) is possible within favourable forest areas in Central Africa. We argue that fragmentation of the existing Pygmy populations, alongside pressure from extractive industries and sometimes conflict with conservation areas, endanger their future. There is an urgent need to inform policies that can mitigate against future external threats to these indigenous peoples’ culture and lifestyles.

## Introduction

Locational information and population estimates are crucial for developing appropriate human rights and land security safeguards for indigenous peoples [1]. However, there are considerable challenges to evaluating numbers or their actual geographic ranges. In the case of the Pygmies (see S1 File), there is uncertainty on current numbers living in Central Africa. For example, there may be between 100,000 and 250,000 Pygmies in DRC as a whole, though some estimates mention up to 660,000 [2]. The main difficulty in estimating Pygmy population numbers in DRC is the lack of proper census data, but some approximations are available from censuses for CAR and Gabon, though only households in villages but not forest camps. In the CAR, only 0.3% of the population are likely to be Pygmies, in Gabon the percentage is also well below 1% [2]. Pygmies are thus a small minority in the countries in which they live, politically insignificant, but a central component of national culture and history. Despite small numbers by modern standards, they are the largest group of hunter-gatherers in Africa, and possibly the world.

The geographic distribution of Pygmies in Central Africa has been represented in a number of published maps [3–6]. Although there are some coincidences between these maps, they are imprecise because they have relied on unverified range approximations from verbal or informal reports of field workers. Published distribution ranges of the various Pygmy groups are therefore difficult to compare. Generally, three main groups of Pygmy populations are recognised [3], each containing different ethnic groups: 1) a Western group composed of the Gyeli, Bongo, Kola, and Zimba, inhabiting the western Atlantic forest; 2) the BaYaka (Aka, Luma, Mikaya, Mbendjele, Ngombe and Baka) who inhabit forest west of the Congo River towards the Atlantic coast and speak Bantu and Ubangian languages; 3) Twa (Tua, Toa, Cwa, Boone, Langi, Chua, and many others) living east from the Congo River to Burundi and Rwanda and speak a wide diversity of languages, and 4) an Eastern group, the Mbuti (Efe, Asua, Sua and Kango), living in the Northeasternmost part of the Central African belt in the region of the Ituri rainforest and Lake Victoria, who speak Bantu and Central Sudanic languages.

Although accumulated knowledge on culture and lifestyles of Pygmies has increased in recent years [7], no one has attempted to predict the occurrence or areas of ecological importance for the largest groups of remaining active hunter-gatherers in the world. Here, we employ a species distribution modelling (SDM) technique [8] based on the favourability function [9, 10], to forecast the distribution of Pygmies in the Congo Basin. Favourability is a measure of the degree to which local conditions lead to a local probability higher or lower than that expected at random, being this random probability defined by the overall prevalence of an organism [9]. SDM techniques have been utilised to predict contemporary [1, 11] and Palaeolithic [12] human populations.

## Materials and Methods

### Study Area

**Fig 1:**
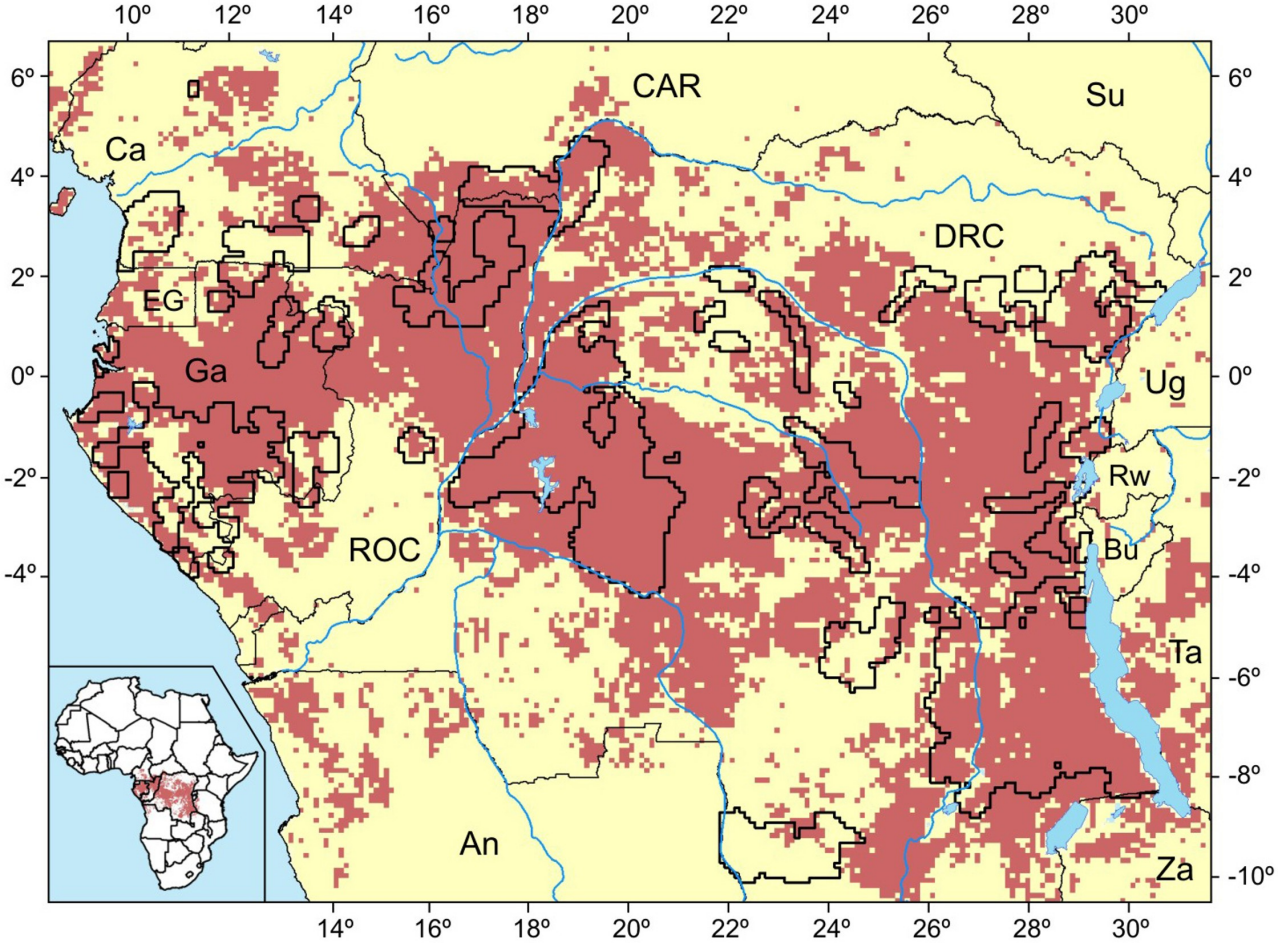
Environmental favourability (F) model for Pygmies. Red: F > 0.5; yellow: F < F. Presence areas are delimited with a thick black line. (Ca: Cameroon; CAR: Central African Republic; Su: Sudan; EG: Equatorial Guinea; Ga: Gambia; RoC: Republic of Congo; DRC: Democratic Republic of the Congo; Ug: Uganda; Rw: Rwanda; Bu: Burundi; Ta: Tanzania; An: Angola; Za: Zambia).

Pygmy groups are found in forests within the limits of the Rainforest Biotic Zone (RBZ) of central Africa. The RBZ, as defined by Happold & Lock [13] encompasses six main countries (DRC, ROC, CAR, Cameroon, Gabon and Equatorial Guinea), as well as parts of another three (Angola, Burundi and Rwanda) (Fig 1). The main vegetation type in the region is Guineo-Congolian lowland rainforest, concentrated in the Congo basin, corresponding to the second largest (close to 2 million km^2^) and the least degraded area of contiguous tropical rainforest in the world. These forests constitute almost 91% of Africa’s rainforests—they are the continent’s main forest resource and home to an extraordinary biological and cultural diversity.

### Pygmy occurrence data

We gathered georeferenced location data (S1 Table) for a total of 654 documented Pygmy camps (S1 Fig) in five Central African countries (Cameroon: 240, CAR: 76, Gabon: 82, ROC: 39, DRC: 217) available for our study. All camps considered in this study were of Pygmy groups only, since Pygmies are intermixed with Bantu families in some villages. These data, derived from field observations of Pygmies in forest during 1985–2014 (though >75% were post-2008), were treated as definite presence of Pygmies at the time of each field study. Registered localities were a mixture of ancestral areas, as well as sites used by Pygmies after displacements by other ethnic groups [14] and forced relocation and sedentarisation [15,16].

We supplemented the more precise Pygmy camp information with published extent-of-occurrence maps of Pygmy distributions for Gabon [17] and DRC [18]. These maps contained distribution polygons within which Pygmy occurrence had been established through national consultations with Pygmy support organizations, representatives of the government and donors.

Although the interplay of social structure, environmental conditions, and cultural factors affect hunter–gatherer population size and demography, there is evidence that similar factors to other mammals, condition how human societies interact with their resource base. Generally speaking, area used and population size of human settlements is positively correlated [19]. In the case of Pygmies, according to data for measured subsistence areas (*n* = 29 camps, S2 Table), published in Hoare [20], average camp size was 248.03 ± 43.95 people (mean ± SE, range 12–842) and mean subsistence area was 1,079.38 ± 98.0 km^2^ (range 214–5,964 km^2^). Population sizes and subsistence areas were significantly positively correlated (*R*^2^ = 0.14, *P* < 0.05).

Although there is still a scarcity of data on Pygmy movement ecology and space use, territory sizes are unlikely to be circular, because Pygmies, like most human resource users, are central place foragers [21]. Therefore, movements for hunting and foraging away from settlements are likely to be linear, consisting of an outward journey, a period of resource extraction and a return journey 34]. Recorded maximum travel distance is almost 76 km [22] although the average from 36 studies is 21.0 ± 3.65 km [20,22–25], varying by group and possibly habitat. Among Aka Pygmies, movements between 0–20km and 60–80km have been recorded [24].

For the purpose of this study, we generated a theoretical unit area of land around each known Pygmy camp liable to be exploited for natural resources. To determine this area, we first determined the mean radius (18.5 ± 1.0 km) encircling a settlement, by using average subsistence area. From this, we created a buffer zone of 20-km, on the basis of the mean radius calculated and on the average travel distance of 21 km (see above). We then applied this buffer to all camps in our database to plot onto a 0.1° × 0.1° map of the study area (6.7°N, 10.5°S, 31.6°E, 8.4°E). This resulted in 5,926 grid cells of Pygmy presence, out of the total of 35,340 cells that covered the entire study area (Fig 1). With this grid approach, which is equivalent to a systematic sampling that covered the whole extent of the study area, we aimed at minimizing bias outcomes resulting from spatial dependence among observations (i.e. autocorrelation [26]). We then considered absences to be those grid cells not included in the presence-grid-cells subset, and used the Pygmy presences/absences for modelling environmental favourability.

### Distribution modelling of environmental favourability

To model the potential distribution of Pygmies throughout Central Africa, we used the Favourability Function [9]. The Favourability Function is based on logistic regression, but cancels out uneven proportions of presences and absences in the modelled data. Favourability thus assesses the extent to which the environmental conditions change the probability of occurrence of an organism with respect to its overall prevalence in the study area. Here, we take this approach to model the relationships between human societies and environmental variables.

We first built an environmental favourability model for Central Africa, by considering 34 predictor variables (S3 Table). Ecological factors that could condition environmental favourability for hunting and gathering were based on habitat descriptors such as climate, topo-hydrography and ecosystem type. Alongside these, we included descriptors of human land use and human activity to represent anthropogenic impacts.

We employed a combination of five climate variables (*maximum annual temperature*, *minimum annual temperature*, *maximum annual temperature range*, *annual precipitation*, *and intra-annual pluviometric irregularity*), four topo-hydrographic indicators (*elevation*, *slope*, *distance to water masses*, *and distance to minor rivers*) as well as 8 ecosystem type descriptors based on vegetation structure (*broadleaf evergreen/semideciduous rainforests*, *swamp forests*, *deciduous forests*, *woody savannas*, *shrublands*, *grasslands*, *deserts*, *intact forest*). Additionally, we considered 17 indicators of anthropogenic activity in terms of human concentration (*rural population density and distance to populated places*), infrastructures (*distance to roads and distance to railroads*), agriculture (*intensive croplands*, *non-intensive croplands*, *cropland [>50%]/vegetation mosaics*, *vegetation [>50%]/cropland mosaics*, *global constraints for cropping activities*, *and percentage of area equipped for irrigation*), livestock (*pasture and browse*, *density of poultry farms*, *density of pigs*, *density of cattle*, *and density of small ruminants*), nature conservation policies (*distance from protected areas*), and exploitation of fauna (*bushmeat extraction*). All variables defining types of land-cover/use were computed as cover percentages in every grid cell, and the rest of variables were estimated by averaged grid-values. All spatial operations, including the calculation of distances to water flows, infrastructures and populated places, were performed using ArcGIS 10.0.

We excluded nonlinear and interaction effects from the model, in order to keep its mathematic formulation as simple as possible for explanatory purposes. To account for Type-I errors caused by the large number of variables considered in our analyses, we controlled the False Discovery Rate (FDR) [27]. Thus, using the presence/absence of Pygmy settlements as the dependent variable, we ran a logistic regression on each of the 34 predictor variables, and only significant (*P*< 0.05) variables under an FDR of *q* < 0.05 were accepted as part of a multivariate environmental model. Only then did we perform a multiple logistic regression employing forward stepwise variable selection (using IBM SPSS statistics 22), and finally transformed probability outputs into favourability values [9, 10].

The model was finally assessed for calibration using the Hosmer-Lemeshow [28] index and the Rooted Mean Square Error (RMSE) [29]; for discrimination capacity using the Area Under the receiver-operating-characteristic Curve (AUC) [30]; and for classification capacity using sensitivity, specificity, Cohen’s Kappa [31], and under- and overprediction rates [32]. For calibration purposes, we used 10 probability bins based on equal distribution of presences; classification measures were based on the 0.5 favourability threshold, because probability is equal to the overall prevalence at this level [10]. Classification and discrimination capacities were expected to be moderate, given the scattered and incomplete knowledge of Pygmy distribution, and the differing underlying causes affecting site selection by Pygmies. Despite this, we still expected to find a favourability model significantly explained by predictor variables, well calibrated, and discriminative.

### Explanatory analysis of variables in the model

We employed a variation partitioning procedure to measure the relative participation of three factors [macroecological indicators (i.e. climate), habitat descriptors (i.e. topo-hydrography and ecosystem types) and anthropogenic influences] on the model explanation of favourability for Pygmy occurrence [26,33]. In this way, we specified how much of the variation in favourability was accounted for by the pure effect of each factor (i.e., variation that is not affected by covariation with other factor), and what proportion was clearly attributable to more than one factor (i.e. shared effect).

The significance of the influence of all variables in the model was assessed using the univariate Wald test statistic [28]. Stepwise methods tend to select variables acting on a larger scale in the first steps and add at subsequent steps only variables significantly related to the residuals not accounted for by previously incorporated variables [34]. The regional relevance of every variable was, thus, analysed using two approaches. Firstly, we measured the correlation (Spearman *R*) of each variable with the favourability output, and compared the sign of *R* (which indicates global relationship within the study area) with the sign of the variable coefficient in the model equation (which indicates the sign of the variable contribution to explaining favourability). Secondly, we visualized the regional contribution of each variable to the model by mapping the difference between favourability values obtained in successive steps, along the stepwise variable selection.

### Estimating Pygmy population densities

VanDerWal *et al*. [35] have suggested a positive association between environmental favourability values and population size. Empirical evidence for a large number of vertebrate species shows that local population density is positively related to environmental suitability [36]. This relationship is expected to be triangular, since many factors may reduce the theoretical maximum density that a species can reach at a certain location. Favourability, in particular, has been shown to reflect maximum density better than probability when prevalence is uneven, as is the case in this study [37].

The relationship between Pygmy population density and environmental favourability was examined in 90 grid cells (*n* = 188 localities) for which camp-size data were available (S1 Table, S1 Fig). We calculated Pygmy population densities from the sum of all Pygmy population figures reported for the same 0.1° × 0.1° grid cell (123 km^2^ at the Equator). Coverage of existing camps for the study areas included here is likely to be fairly complete (given the manageable size of the grid cell used). However, two caveats exist: first, camps varied in their dependence on forest resources, i.e. all hunted and gathered, but some relied upon farming more than others; and second, not all reported population sizes were taken during the same time period. We examined the shape of the population—favourability values point cloud, after population-size outliers were eliminated following Tukey [38] [i.e., if population size > Q3 + 1.5 × (Q3—Q1), where Q1 and Q3 are the first and the third quartiles, respectively] and found a typical wedge-shaped relationship. We were then able to use ordinary linear regression to test the significance of a positive relation, and a quantile regression [39] to extrapolate the upper limit of population size to the whole study area, as a function of favourability.

### Estimating Pygmy populations

We calculated the Pygmy metapopulation in Central Africa. We use the term metapopulation here to encompass all spatially separated populations of Pygmy groups, which may interact at some level. First, we divided the range of environmental favourability into three distinct categories, unfavourable: <0.2, medium: 0.2–0.5, favourable: >0.5. We then calculated the average Pygmy population size empirically observed in all 123-km^2^ grid cells with favourability values belonging to the three categories; these averages were built upon the 90 grid cells for which data on settlement size were available (excluding outliers). Using these figures, we then calculated the potential population size (*PPS*) for every grid cell in the study area, according to their favourability values. Finally, we summed all *PPS* values for the entire study area, but applied the following correction to take territoriality into account:

Metapopulation=GPPS×GCS/ASA (1)

where the metapopulation is the net potential population size; *GPPS* is the gross potential population size resulting from the sum of the *PPS* values; *GCS* is the size of a grid cell (i.e 123 km^2^); and ASA is the average subsistence area estimated for Pygmies (i.e. 1,079 km^2^, see Pygmy Occurrence Data above). The resulting metapopulation number was estimated for the entire Central African study area, and then individually computed for each of the eight countries in which Pygmies occur.

### Relationship between Pygmy camps and roads

From colonial to recent times, a large number of Pygmies have been subject to relocations, voluntary and forced, along roads [15]. We analysed the link between environmental favourability and proximity to roads as a means of testing whether Pygmy camps are disadvantaged close to roads. We used box plots to examine the relationship between favourability and distance to roads for those localities where Pygmy presence has been recorded (see above). Favourability was divided into the three distinct categories for estimating metapopulation size (i.e. unfavourable: <0.2, medium: 0.2–0.5, favourable: >0.5). We used an analysis of variance (ANOVA) to determine whether differences between the three classes are significant, for the log-transformed distance values [40]. Tukey’s honestly significant difference (HSD) post hoc tests were applied when significant differences (p < 0.05) were found between the favourability classes.

## Results

We obtained a significant environmental favourability model for Pygmy distribution in Central Africa (Fig 1 and S1 Fig). The model was significantly well calibrated (Hosmer-Lemeshow index = 14.18; *P*_10_ > 0.05), with a low deviation between observed and predicted presences (RMSE = 49.9; for a total of 5926 presences). The model had an acceptable discrimination capacity (AUC = 0.770), and a fair classification capacity (Cohen’s Kappa = 0.246). The proportion of correctly classified presences was higher than that of absences (sensitivity = 0.733; specificity = 0.659), meaning that favourable areas covered recorded presences but were not restricted to them. Although under-prediction was low (0.073), meaning that Pygmies were observed to occur in a low proportion (7%) of unfavourable areas, the model had a high over-prediction rate (0.704), thus indicating that Pygmy presence was not reported in 70% of favourable areas.

Our favourability model combined 26 of the 34 proposed variables to explain significantly the presence of Pygmies in 5,926 of the 35,340 cells (Table 1). Habitat descriptors and anthropogenic influences explained a similar proportion of the spatial variation in favourability: 54.5% and 62.6% respectively (S2 Fig). However, climate only explained 23.4%. Shared effects between different factors (meaning either cross or indistinguishable explanatory power) were found; habitat and anthropogenic factors shared 26.2% of their influence, whilst climate shared 14% with the other two factors. As a result, the pure effect of habitat and anthropogenic influences respectively only explained around 30% of favourability each. The pure effect of climate explained less than 10%.

**Table 1:** Descriptor variables of the environmentally favourable areas for Pygmies according to the favourability model. Step: Order or entrance in the model; W: Univariate Wald test statistic quantifying variable significance in the model (all the variables shown were significant with *P* < 0.05); CfS: Sign of the variable coefficient in the model; CrS: Sign of the correlation (Spearman) between the variable and favourability values; AGI: Area of geographic influence of the variable in the model within the Central African study area (N = North, S = South, E = East, SE = South-East, W = West).

Variable	Step	W	CfS	CrS	AGI
**Variables describing favourable areas for Pygmies globally**					
Rainforest	3	499.3	+	+	
Distance to water masses	2	420.3	-	-	
Flooded forest	5	269.8	+	+	
Distance to railway	12	232.7	+	+	
Distance to roads	1	193.4	+	+	
Min. temperature	22	29.7	+	+	
Bushmeat extraction	17	25.1	-	-	
Intact forest	19	19.7	+	+	
Constraints for agriculture	20	12.6	-	-	
**Variables describing favourable areas for Pygmies regionally**					
Deciduous forest	4	482.5	+	-	E, SE
Cropland	9	372	+	-	N, E, SE
Woody savanna	6	288.1	+	-	N, E, SE
Rainfall seasonality	13	137.2	+	-	E, SE, W
Veg./crop mosaic	14	128.4	+	-	E, SE
Herbaceous vegetation	15	50.7	+	-	W
Altitude	21	43.2	+	-	E, SE
Sheep/goat prod.	24	12	+	-	N, E, SE
**Variables apparently outlining unfavourable areas for Pygmies**					
Beef production	8	211.4	-	-	
Distance to populated places	7	144.4	-	+	
Temperature range	11	141.6	-	-	
Precipitation	10	80.8	-	+	
Poultry production	16	63.1	-	-	
Rural population density	18	23	-	-	
Irrigation equipment	23	14.3	-	-	
Pork production	26	11.1	-	-	
Agricultural land	25	4.7	-	-	

Variables with the highest explanatory power within the model (Wald statistic > 250) were primarily habitat type descriptors (Table 1). In fact, the correlation (Spearman *R*) between favourability and forest surface area (i.e. the sum of *broadleaf evergreen/semideciduous forests*, *swamp forests* and *deciduous forests*) is highly significant: *R* = 0.667, *P*_35, 341_ < 0.001 (S3 Fig). Broadly speaking, habitat variables, and descriptors of the intersection between farming and forest areas, were positively related to high environmental favourability areas for Pygmies. In contrast, most anthropogenic variables limited the environmental favourability for Pygmies in the model (*livestock*, *indicators of intensive agriculture*, *and communication infrastructure*). Some variables, such as *broadleaf evergreen/semideciduous rainforests*, *swamp forests*, *short distance to water masses*, *and long distance to roads and railroads*, had a positive influence on the presence of Pygmies, i.e. they showed identical signs within the model equation and in the variable correlations with the model, and their entry into the model produced a generalized increase of favourability (Table 1, S4 Fig). Other variables [*deciduous forests* and *vegetation/cropland mosaics* (East/South-East), *woody savannas* and *non-intensive croplands* (also in the North), *annual pluviometric irregularity* and the *distance to populated places* (also in the West)] also had positive influences, though they were limited to a regional context, mostly to the east and the south-east (i.e. the sign within the model equation was positive, whereas that of the correlation with the model was negative; and their entry into the model produced a regional increase of favourability). Finally, a set of variables, most of them indicators of farming practices (livestock and intensive agriculture), represented environmental constraints to Pygmy occurrence, especially in the peripheral areas (i.e. both signs within the model equation and of variable correlations with the model were negative; and their entry into the model produced a regional decrease of favourability).

**Fig 2:**
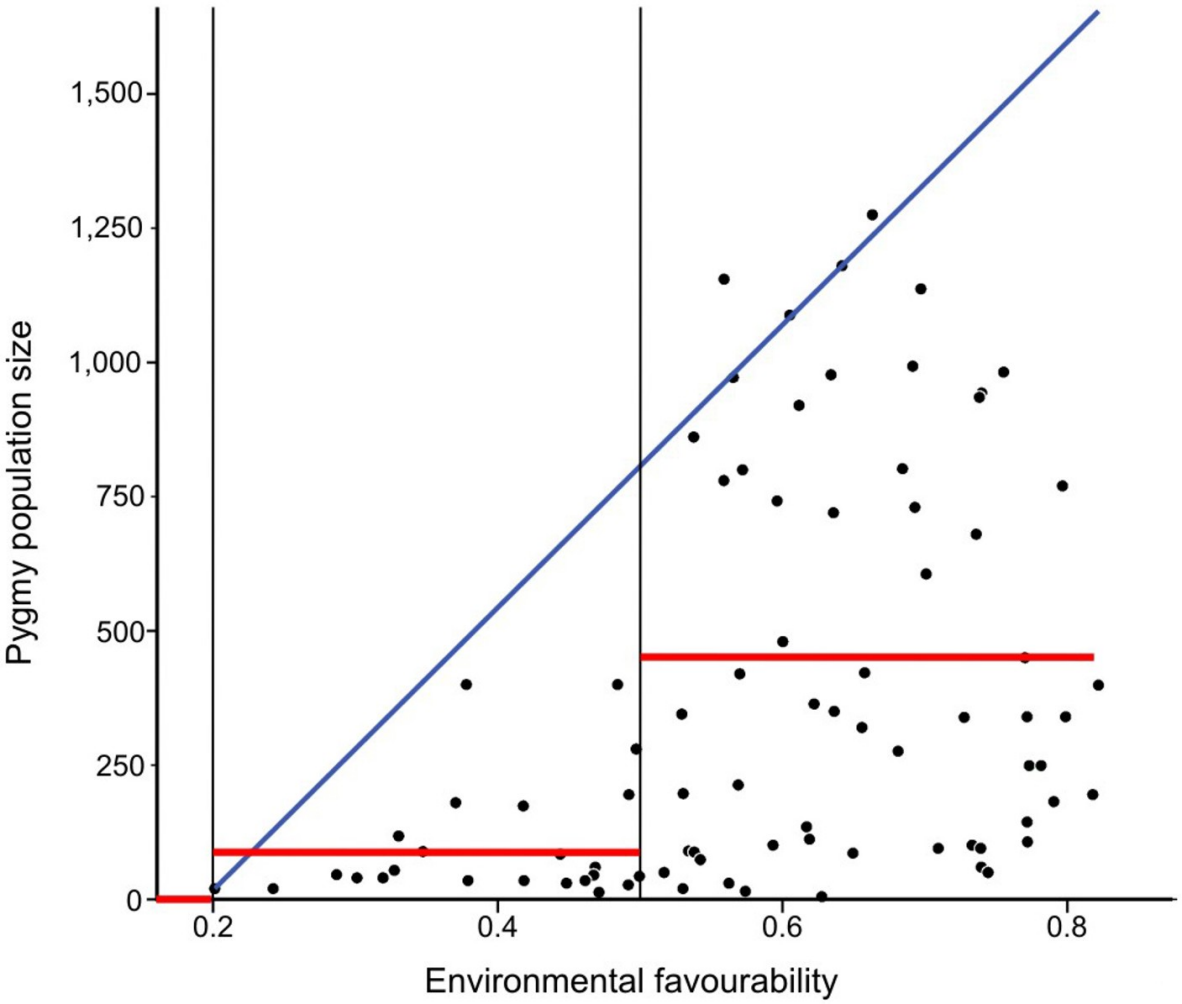
Space defined by predicted environmental favourability (x-axis) and population size (y-axis). The scatter plot shows a polygonal wedge-shaped spread of points with the upper limit increasing at higher favourability values. The blue line fits the quantile regression with the 95th percentile, representing the upper limit of potential population size. Red lines indicate average population size considered, in every favourability category (<0.2, 0.2–0.5, >0.5), for estimating net potential population size in the Central African studied area.

Predicted environmental favourability (x-axis) and population size (y-axis) exhibited a polygonal wedge-shaped spread of points with the upper limit increasing at higher favourability values (Fig 2); five outliers (population size > 1,563 inhabitants / km^2^) were eliminated from the dataset. Population size and environmental favourability were positively correlated (*F*_1,82_ = 13.3, *P* <0.001). The quantile regression with the 95th percentile outlined very narrowly the upper limit of population size as a function of favourability: Upper limit of population size = -509.767 + 2,633.026 × Environmental Favourability (see Fig 2 and S1 Fig).

**Fig 3:**
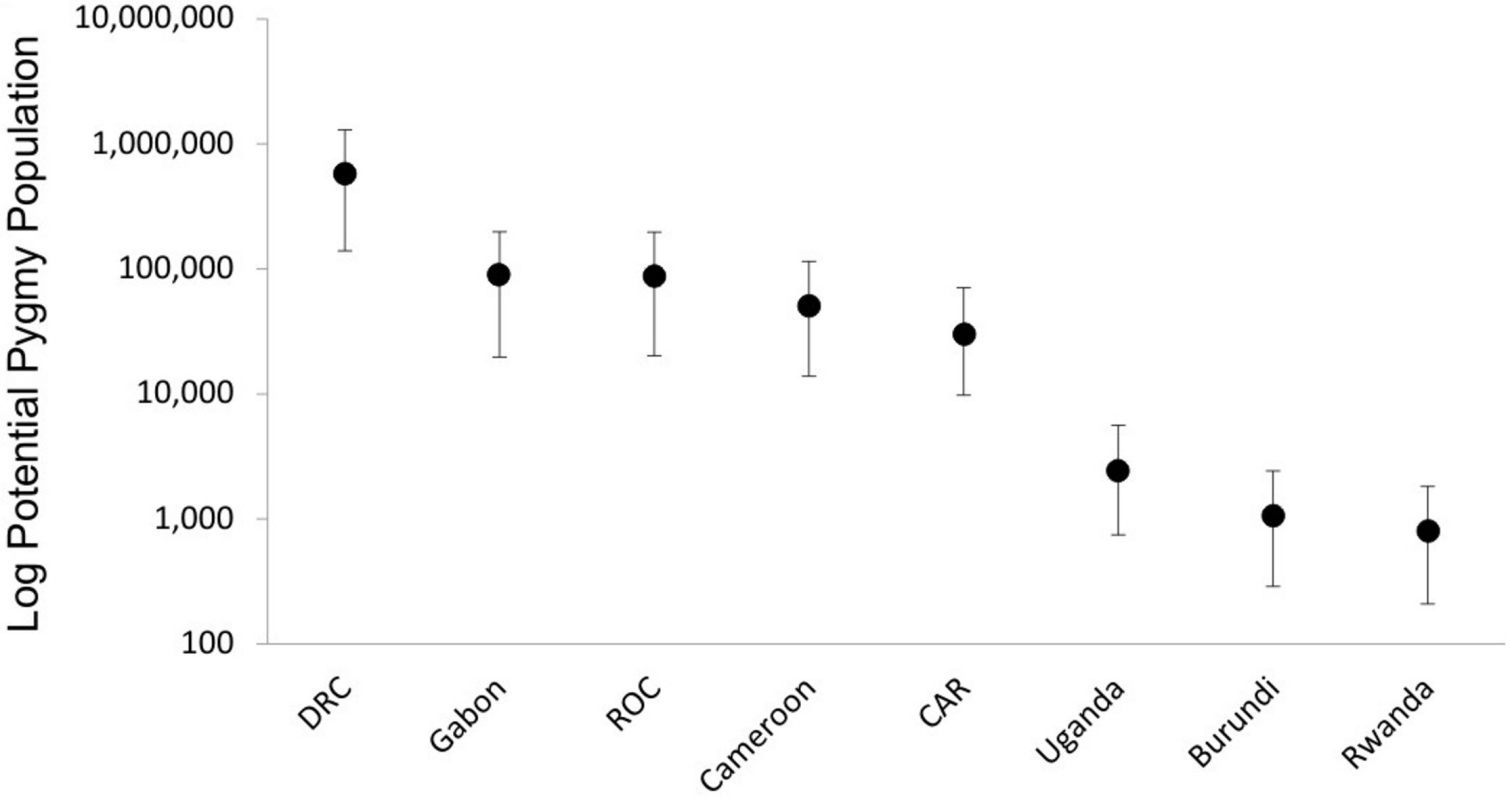
Estimates of potential Pygmy population size by countries in Central Africa.

We calculated a Pygmy potential metapopulation size for Central Africa of 919,500 ± 226,500. By country, the largest potential population of Pygmies was estimated for the DRC, followed by Gabon, ROC, Cameroon and CAR; the smallest populations were for Rwanda, Burundi and Uganda (Fig 3).

**Fig 4:**
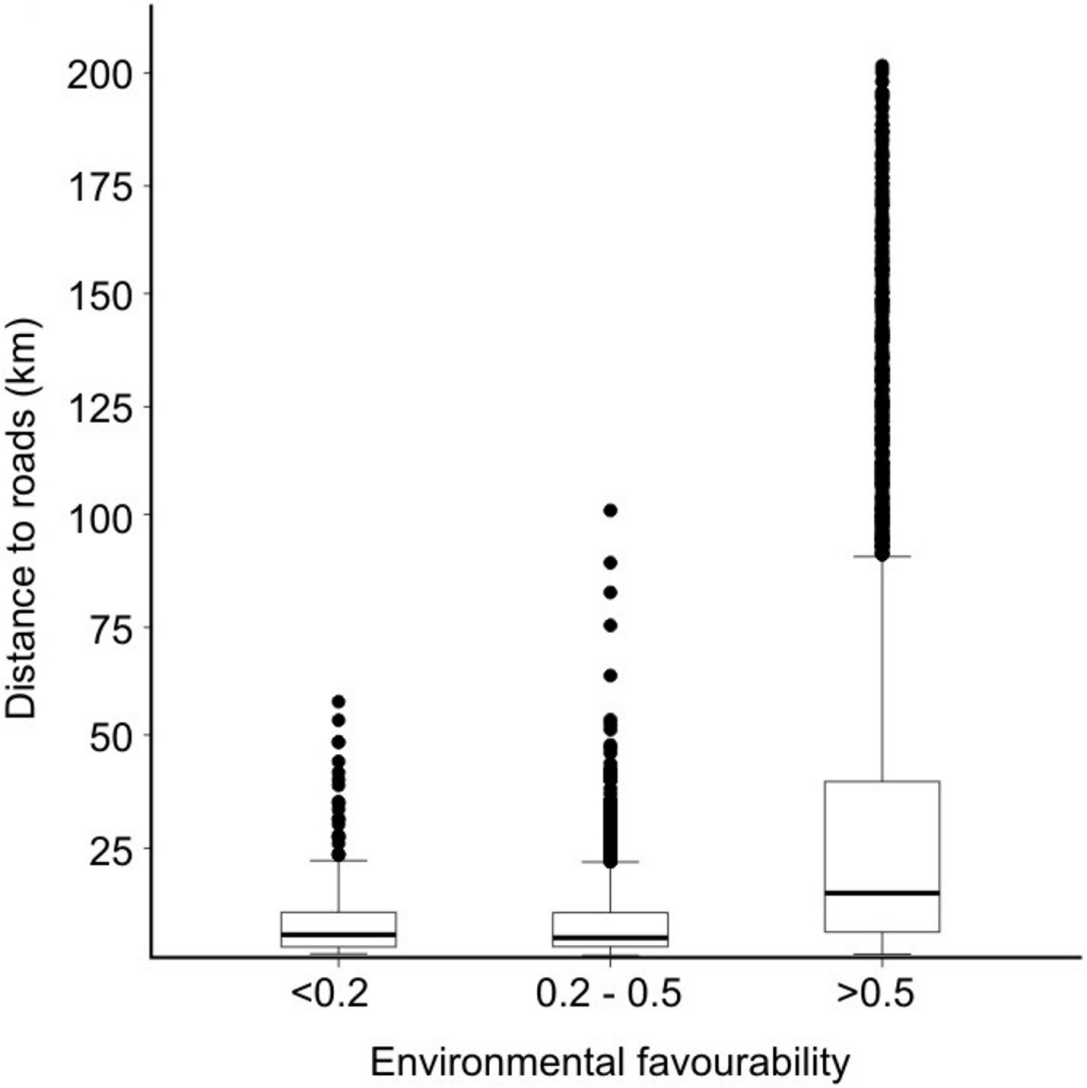
Box-plot showing relationships between favourability and distance to roads, within the areas recorded as Pygmy presences. Box upper limit: Q3; box lower limit: Q1; horizontal line: median; whisker limits: Q1-1.5×(Q3-Q1) and Q3+1.5×(Q3-Q1); points: outliers.

Distance to the road network was significantly related to environmental favourability (ANOVA: *F*_2, 5923_ = 262.5, *P* < 0.001; Fig 4). This distance was significantly higher in the most favourable areas (favourability value > 0.5) compared to unfavourable (< 0.2; HSD = 21,833.3; p < 0.001) and intermediate-favourability areas (0.2–0.5; HSD = 23,309.4; *P* < 0.001).

## Discussion

This paper is the first to have compiled such a large collection of known location and population data of Pygmy camps in Central Africa. These records (75% gathered after 2008, and 81% post-2000) allow for a valid comparison given that Pygmy dispersal is strongly localized. Genetic studies of Western Central African Pygmies indicate a strong differentiation, suggesting that in the Baka, dispersal over wide geographical areas rarely occur [41], though population concentration (further travel at lower densities) and culture can alter this (B. Hewlett, pers. comm.). Thus, All things being equal, camp movements, if they did occur between our first (1985) and last studies (2014), were unlikely to have significantly changed the location of the sites to have affected our modelling outputs.

Our model classified potentially suitable areas for Pygmy settlements in a fairly robust manner, despite the relatively sparse data available on Pygmy presence. Although we were able to cover only 17% of the total surface of the study area, we did not find any evidence that our model was biased by overfitting [42]. The most favourable areas for Pygmies according to our model are those areas contained within Guineo-Congolian forests of Central Africa (technically the Congo–Ogooué Basin and contiguous forests, hereafter termed the Congo Basin for brevity), which accounts for 89% of African rainforests. In our model, three forest variables (*broadleaf evergreen/semideciduous rainforest*, *deciduous forests* and *swamp forests*), included among the first five variables entered in the stepwise variable selection procedure, were the most important descriptors of Pygmy presence. In fact, the correlation between the complete favourability model and the 5th step-model (from the first five variables) was 0.783, thus confirming that all variables other than the five main ones were of lower significance. Hence, rainforest variables had a wide-scale positive influence on Pygmy presence. This relationship is strong, not just in those countries where we were able to obtain direct camp data, but also in those others (Rwanda, Burundi, Uganda) where Pygmies also occur [7] but for which no location data were available.

Our model also indicated that *deciduous forests* were important for determining Pygmy presence, but this biome is limited to the east and the south of the Congo Basin. This region, primarily the east (Nord and Sud Kivu) and Southeastern DRC (North of the Katanga province), has experienced one of the most intensive sedentarisation processes among Pygmies [18]. In Katanga province, most Pygmy populations no longer live in the forest, but are confined to its margins; links to the forest have been nearly or completely severed along the shores of Lake Tanganyika, and conflicts over access to natural resources still occur [18]. In our model, marginal areas of the main rainforest block, i.e. *deciduous forests*, *woody savannas* and *non-intensive croplands*, are the main descriptors of Pygmy presence in the East and the South-east, as well as in the North of the Congo Basin. Our model was, thus, able to discriminate between the principal areas of Pygmy distribution and more marginal habitats.

One of the most significant variables in our model was the *distance to roads* (Table 1 and S3 Table). Distance to roads was significantly greater in those areas that were environmentally most favourable for Pygmies (Fig 4), especially in the central Congo Basin (see Fig 4 and S1 Fig). In contrast, Pygmy settlements in unfavourable areas were largely linked to roads (S5 Fig). This is the first quantitative indication that Pygmy settlements relocated to roadside areas are in environmentally suboptimal conditions compared to favourable areas determined by our model. This observation is not surprising given that roads and other linear clearings can have an array of deleterious effects on tropical forests and their wildlife [43], and particularly in diminishing hunting resources [44]. From a socioeconomic point of view, Pygmy groups that have voluntarily moved away from less impacted forest areas have sought opportunities for work and trade [18], some of them owning fields [45]. Pygmy groups that have been relocated as part of official sedentarisation programs set up by governments may have failed to adjust to the new living conditions, often with severe consequences to their way of life [15,23,46]. Reasons for displacements may range from more indirect causes, such as deforestation for agriculture, logging or mining, to forced displacement under social evolutionary ones, which impose European development models which argue that indigenous groups and the protection of areas for nature conservation are incompatible [15, 23, 47].

Through our population-favourability analyses we estimated a population of around 900,000 Pygmies possible throughout all potential favourable areas in Central Africa, more than 60% in DRC. This figure cannot be verified against any available population census data since we extrapolate to areas outside the known distribution ranges of Pygmies. However, it is highly likely that Pygmy populations occur outside the polygons of distribution used for our study, as inferred from the latest Pygmy distribution map generated by the Rainforest Foundation-UK Mapping for Rights program [35].

Censuses are lacking for almost all groups, and estimations of the main Pygmy populations are generalisations from a few studied settlements or a direct extrapolation to areas presumed as occupied based on unclassified-maps. Thus, direct comparisons between our population estimates and published population figures are difficult, primarily because methods on how actual numbers were calculated for the latter are not explicitly described in the literature. The picture that emerges from published estimates for most groups is one denoting a wide spectrum of circumstances ranging from not more than 400 for the Bedzan in Cameroon, to around 50,000 for the Aka in CAR and ROC [7]. In most cases, the degree of fragmentation of all Pygmy groups is high, perhaps more clearly seen for the Bongo Pygmies where around 3,000 Pygmies may be distributed in about 43 subpopulations in Gabon [17]. More dramatically perhaps, our metapopulation estimate for all groups can be presumed to be relatively low, given the total area in which the close to one million estimated Pygmies are found. The metapopulation of Pygmy groups can be considered, at least in theory, to consist of several distinct populations together with areas of suitable habitat, which are currently unoccupied. Each population cycles in relative independence of the other populations and eventually goes extinct as a consequence of demographic or environmental stochasticity (fluctuations in population size due to random demographic events or to natural catastrophes); the smaller the population, the more prone it is to extinction. Although individual populations have finite life spans, the metapopulation as a whole is often stable because immigrants from one population (which may, for example, be experiencing a population boom) are likely to re-colonize habitat, which has been left open by the extinction of another population. Whether these subtleties of population exchanges are likely to happen to ensure the long-term viability of Pygmies throughout Central Africa is still unknown. Although much progress has been made in understanding dispersal and their implications on genetics of Pygmy groups, knowledge is insufficient at present to understand population connectivity or the impact of expansion of growing populations. On the contrary, the future of all Pygmy groups is severely compromised by threats of morbidity and mortality due to disease, discrimination and marginalisation, social alienation, and conflicts with extractive industries, agricultural expansion and occasionally conservation agendas. The latter may be a source of disagreement that could more easily be resolved, since the inclusion of indigenous peoples in conservation of lands can have more positive impacts on conservation outcomes than excluding them from decision-making [20]. Conservation of tropical forests needs to integrate ecological and cultural components since neither is likely to survive without the other. Because the subsistence economies of indigenous forest peoples are based on the use of and access to natural resources, protection of these resources and of traditional practices for their use, management and conservation are essential to ensure their survival [48]. A number of conventions and similar instruments [49], specify that indigenous and tribal peoples have the right to participate in the use, management, protection, and conservation of natural resources, as well as the right to be asked for their free, prior and informed consent before natural resources on their territories are explored or exploited [50]. Enforcing these already existent instruments is fundamental to ensuring the survival of all Pygmy groups in Central Africa, and that of a unique set of human cultures.

## Supporting Information

S1 Fig Environmental favourability (F) model for Pygmies and correspondence with upper limit of potential population size (UPPS). Presence areas are delimited with a thick black line, and are derived from combining location data with extent-of-occurrence maps. Points indicate location data (grey coloured points had information about population size), which were surrounded with 20-km buffers representing a estimation of subsistence area. Slim black lines correspond to country boundaries. Grey lines represent the road network [Vector Map Level 0 at the Digital Chart of the World (DCW, http://worldmap.harvard.edu), updated in 2002].
(TIF)

S2 Fig Variation partitioning diagram. The numbers specify how much of the variation in environmental favourability for Pygmies explained by the model was accounted for purely by habitat, climate and human factors, and which proportion was attributable to their shared effects (intersections). Values shown are the proportions of variation explained.
(TIF)

S3 Fig Environmental favourability for Pygmies explained by the model. Favourability is plotted against the proportion of forest (i.e. the sum of *broadleaf evergreen/semideciduous*, *swamp* and *deciduous forests*) surface-area in the 35,340 0.1° x 0.1° cells that covered the study area. Green areas in the map represent forests. The blue line represent the lineal adjustment of these points (*R* = 0.667, *P*_35,340_ < 0.001).
(TIF)

S4 Fig Mapped contribution of variables to the favourability model along the stepwise variable selection. Green: positive contribution; red: negative contribution.
(TIF)

S5 Fig Two examples of Pygmy camp locations along the road network, outside the ecologically most favourable areas. (TIF)

S1 File Use of term Pygmy. (DOC)

S1 Table Pygmy-camp data sources. (XLS)

S2 Table Empirical data of territory sizes for Pygmy camps in various localities in Central Africa. Source: Hoare AL. 2007 *Resource rights and timber concessions*: *Integrating local peoples’ land-use practices in forest management in the Congo Basin*. London: Rainforest Foundation-UK.
(DOC)

S3 Table Predictor-variable sources of the 34 predictor variables considered to build the environmental favourability model for Pygmies. (DOC)
