# Supplementary figures and images for: Distribution and Numbers of Pygmies in Central African Forests
Source: PLoS One. 2016 Jan 6;11(1):e0144499. doi: 10.1371/journal.pone.0144499 (PMC4711706; doi:10.1371/journal.pone.0144499)

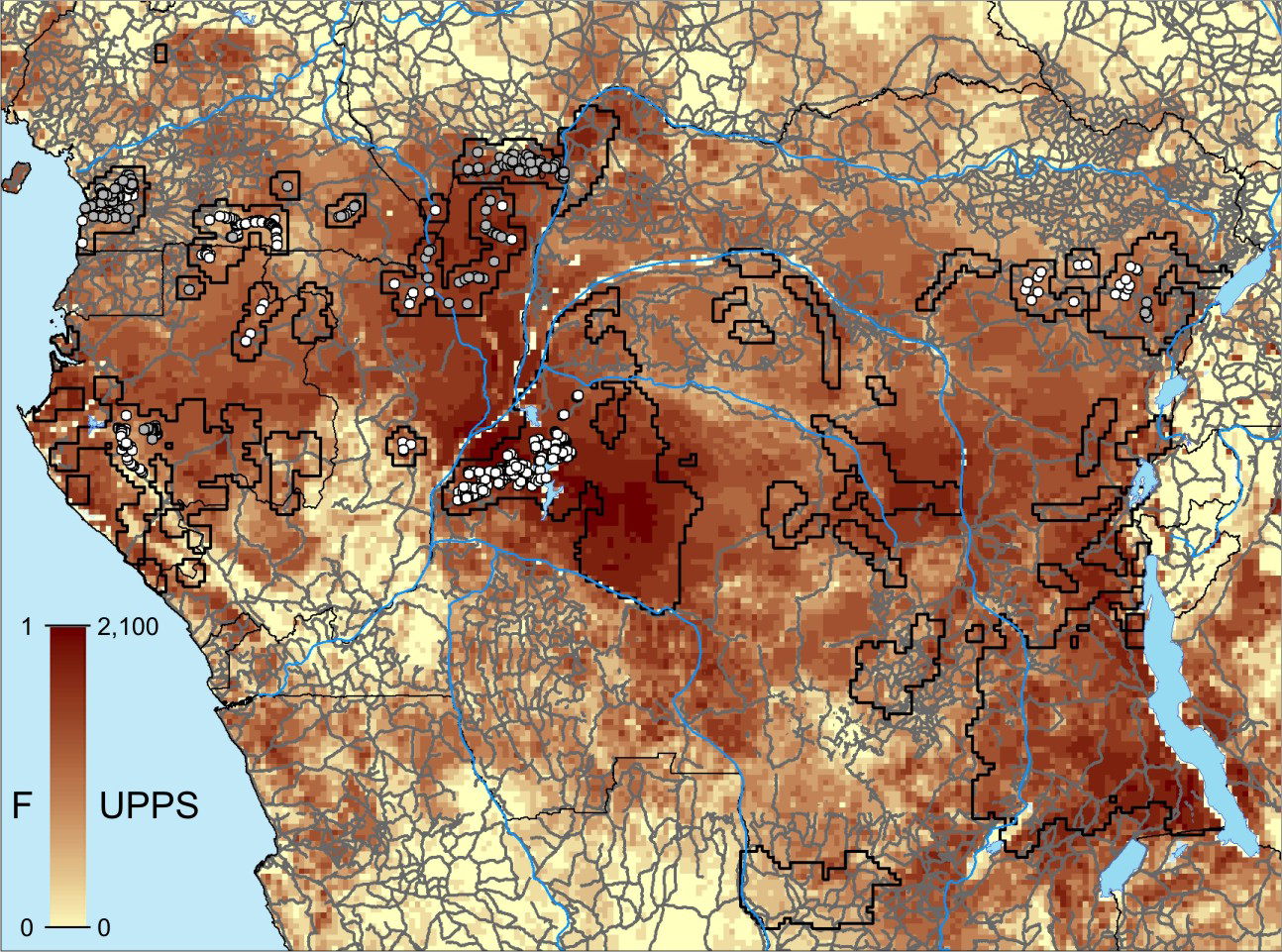

Supplement: S1 Fig — Presence areas are delimited with a thick black line, and are derived from combining location data with extent-of-occurrence maps. Points indicate location data (grey coloured points had information about population size), which were surrounded with 20-km buffers representing a estimation of subsistence area. Slim black lines correspond to country boundaries. Grey lines represent the road network [Vector Map Level 0 at the Digital Chart of the World (DCW, http://worldmap.harvard.edu), updated in 2002]. (TIF) [file pone.0144499.s001.tif]

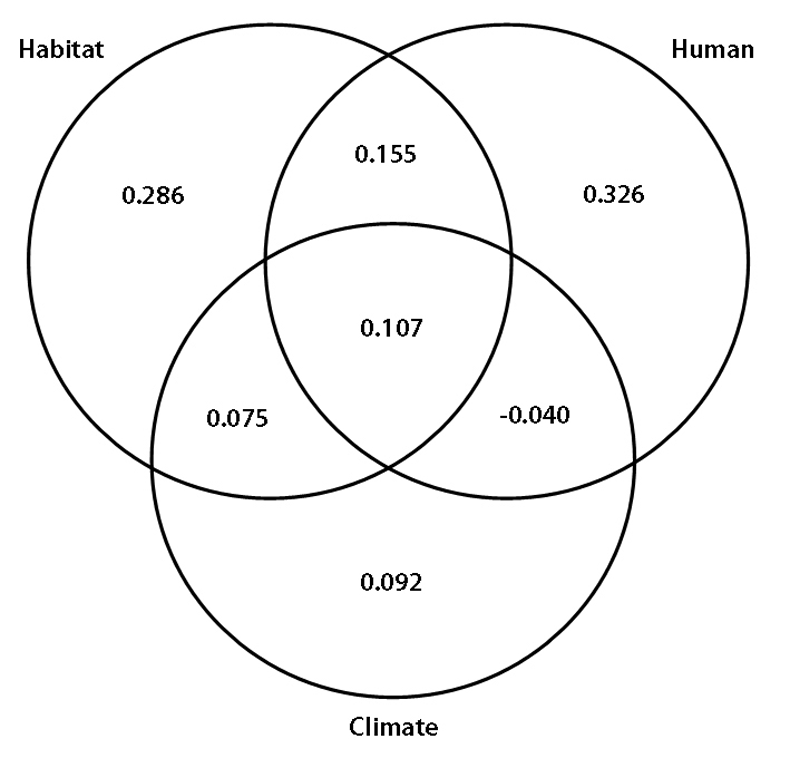

Supplement: S2 Fig — The numbers specify how much of the variation in environmental favourability for Pygmies explained by the model was accounted for purely by habitat, climate and human factors, and which proportion was attributable to their shared effects (intersections). Values shown are the proportions of variation explained. (TIF) [file pone.0144499.s002.tif]

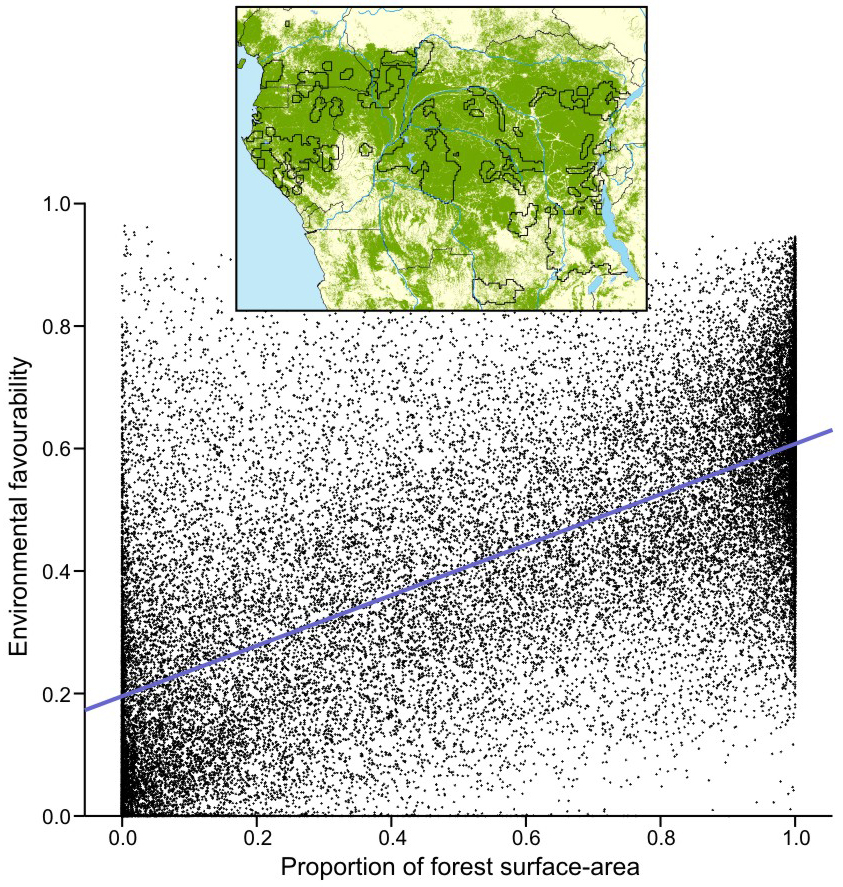

Supplement: S3 Fig — Favourability is plotted against the proportion of forest (i.e. the sum of broadleaf evergreen/semideciduous, swamp and deciduous forests) surface-area in the 35,340 0.1° x 0.1° cells that covered the study area. Green areas in the map represent forests. The blue line represent the lineal adjustment of these points (R = 0.667, P35,340 < 0.001). (TIF) [file pone.0144499.s003.tif]

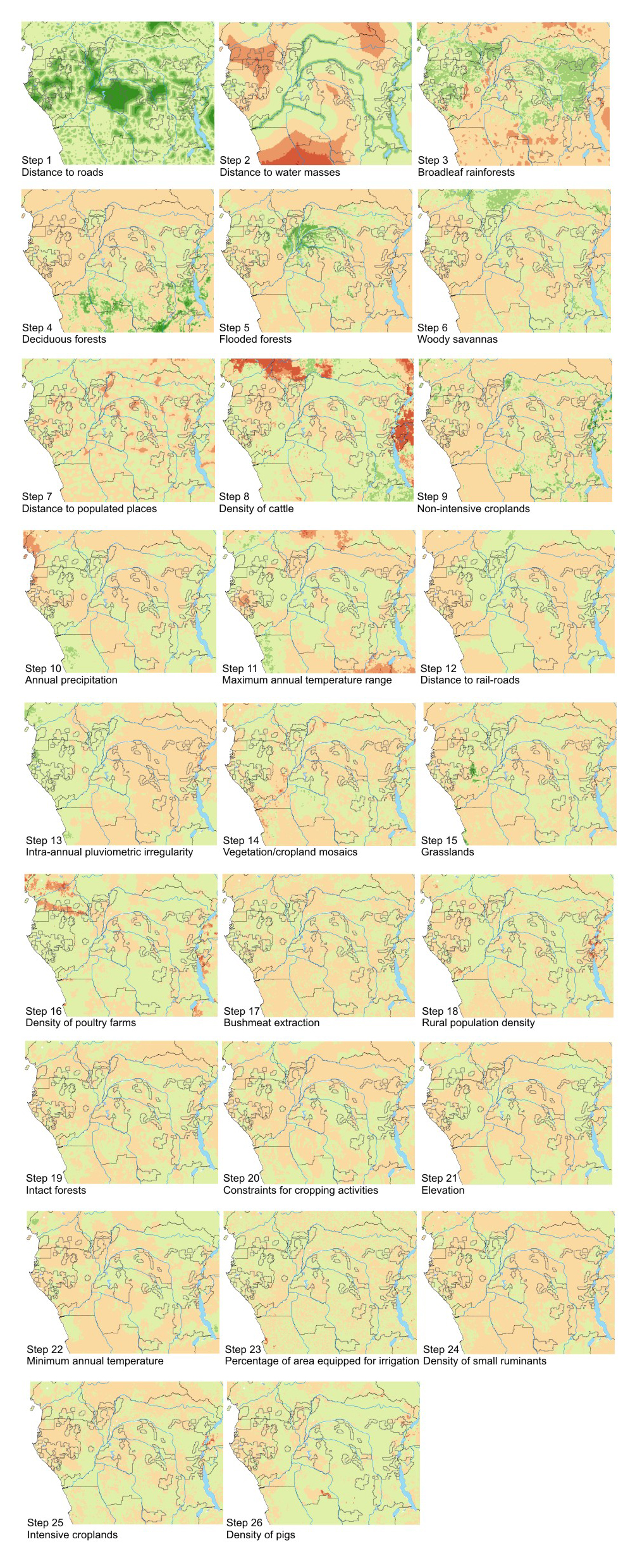

Supplement: S4 Fig — Green: positive contribution; red: negative contribution. (TIF) [file pone.0144499.s004.tif]

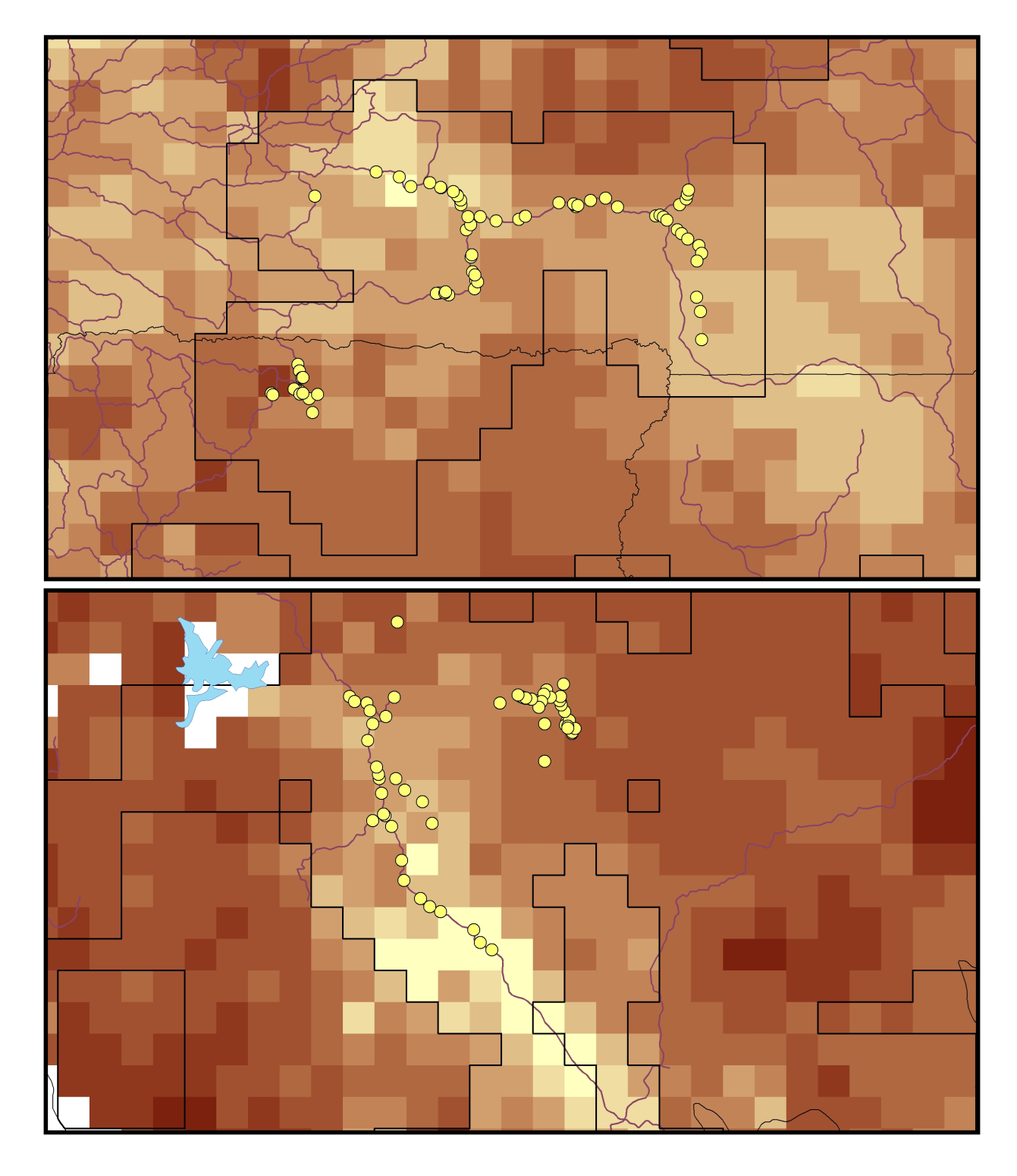

Supplement: S5 Fig — (TIF) [file pone.0144499.s005.tif]
