# Supplementary material for: Distribution and Numbers of Pygmies in Central African Forests
Source: PLoS One. 2016 Jan 6;11(1):e0144499. doi: 10.1371/journal.pone.0144499 (PMC4711706; doi:10.1371/journal.pone.0144499)
Supplement: S1 File — (DOC) [file pone.0144499.s006.doc]

**S1 File**

**Use of the Term Pygmy**

Although numerous alternative terms to Pygmy have been used to refer the rainforest hunter-gatherers of the Congo Basin, none have been agreed upon by academics or the people themselves to replace it. Although some academics and Central African government officers feel the term Pygmy is derogatory or does not adequately represent the people, the term Pygmy *sensu lato*, to refer to all hunter-gatherer groups in Central Africa, is widely used by a broad group of people in Europe, Japan, the United States and Africa. Moreover, International and local NGOs use the term in their titles or literature e.g. *Pygmy Survival Alliance, Forest Peoples’ Programme. Survival International, Rainforest Foundation*, *Reseau Recherches Actions Concerteees Pygmees, Centre d’Accompagnement des Autochtones Pygmees et Minoritaires Vulnerables* and *the Association for the Development of Pygmy Peoples of Gabon*. Congo Basin conservation groups, such as World Wildlife Fund and Wildlife Conservation Society and international human rights groups working in the region, such as UNICEF and Integrated Regional Informaton Networks (IRIN), also regularly use the term Pygmy in the their literature.

In our study, we consider all groups under the umbrella of Pygmy as expressing an equivalent spatial relationship between their presence and their immediate environment. In so doing, we do not ignore the fact that various ‘Pygmy’ groups express distinct cultures and in some case ethnicity from other ‘Pygmy’ groups, and is not meant in any way a disrespect to the various ethnicities. Although it is likely that there may be cultural reasons for geographical location and distribution, we argue that the ecological setting is a primary driver in humans in choosing localities to live in. Ichikawa (2014)14:332-3 – ‘The forest plants and animals provide the people with the basis for their cultural identity. Their life and culture cannot be maintained without the forest in its entirety … The destruction of the forest would result in the deterioration of a culture that is heavily dependent on, and in very significant ways a part of, the Congo Basin rainforest.’

**Background**

Pygmy groups consider themselves, and are judged by their farming neighbours, as the aboriginal people of the Central African forests (Köhler and Lewis 2002). They identify closely with the forest, expressed in the BaYaka saying “A Pygmy loves the forest as she loves her own body” (Lewis 2009), dependent to varying degrees on wild products from the rainforest ecosystem. The archaeological presence of autochthonous Pygmy people in the Congo River Basin is not clear, but recent genetic studies suggest ancestral groups may have entered the forest >50,000 YBP (Patin et al. 2009; Verdu et al. 2009; Verdu 2014). These groups are distributed discontinuously across nine different African countries [Angola, the Democratic Republic of the Congo (DRC), the Republic of Congo (ROC), the Central African Republic (CAR), Cameroon, Gabon, Rwanda, Burundi, Uganda]. Pygmies were also found in Equatorial Guinea (locally known as the Bayele though part of the Kola group of Pygmies from Cameroon), but are now limited to a single extended family in Ayamiken, Northeastern Rio Muni, Equatorial Guinea (Appiah and Gates 2010). Diverse ethnolinguistic Pygmy communities are often hunter-gatherers or former hunter-gatherers, with variable access to wild forest resources today. They trade with neighbouring farmers to acquire cultivated foods and other material items; it is today rare for groups to live isolated in the deep forest without access to agricultural products, though some still do (Lewis 2005). Despite the great diversity of situations Pygmy groups share some remarkable similarities. In particular, an egalitarian social organization bound up in a matrix with other key cultural practices; some of these as spending at least 4 months a year hunting and gathering in the forest; strongly identifying with and preferring forest life; contrasting the “forest world” to the “village world”; having economies based on demand-sharing; practicing important rituals associated with elephant hunting; having intimate parent-child relations; and diverse relationships with neighbouring farming groups (Hewlett 1996). Most Pygmy groups are mobile within a delimited territory or group of territories to which they have affiliations through clan or marriage relations, moving between places for both social and nutritional reasons. Recent legislation in some countries has recognized the rights of autochthones (indigenous or first peoples) (Republique du Congo 2011). However despite such provisions under law, in all countries where Pygmies are found, they are increasingly marginalised, and threatened by disease, displacement, forced sedentarisation, and deforestation (ohenjo et al. 2006; Wodon et al. 2012).

**Data Availability**

Data associated with the environmental favourability model has been uploaded as part of the supporting materials. Due to ethical restrictions, primary data, including the coordinates of Pygmy settlements in Central Africa, are not deposited publicly. For further enquiries contact J. E. Fa (jfa949@gmail.com), Division of Biology and Conservation Ecology, School of Science and the Environment, Manchester Metropolitan University, Manchester M1 5GD, UK.

**References**

Appiah KA, Gates HL. 2010 *Encyclopedia of Africa*. New York: Oxford University Press.

Hewlett BS. 1996 Cultural diversity among African Pygmies. In *Cultural diversity among twentieth-century foragers: an African perspective*. (ed Kent S.), pp. 215–244. Cambridge: Cambridge University Press.

Ichikawa M. 2014 Forest conservation and indigenous peoples in the Congo Basin: New trends toward reconciliation between global issues and local interests. In *Hunter-gatherers of the Congo Basin: Culture, history and biology of African Pygmies*. (ed Hewlett BS), pp. 321-342 – 31-58. New Brunswick (US) and London (UK): Transaction Publishers.

Köhler A, Lewis J. 2002 Putting hunter-gatherer and farmer relations in perspective: a commentary from Central Africa. In *Ethnicity, hunter-gatherers, and the ‘other’: association or assimilation in Southern Africa?* (ed Kent S.). Washington: Smithsonian Institute.

Lewis J. 2005 Using Avoidance to maintain Autonomy. The Mbendjele Yaka of Congo-Brazzaville. In *Indigenous Peoples, their struggles and rights*. (ed Fonseca H.), pp. 75-78. Uruguay: World Rainforest Movement International Secretariat.

Lewis J. 2009 As well as words: Congo Pygmy Hunting, Mimicry and Play. In *The cradle of language, Volume 2: African perspectives*, (eds Botha R, Knight C), pp 232 – 252. Oxford: Oxford University Press.

Ohenjo N, Willis R, Jackson D, Nettleton C, Good K, Mugarura B. 2006 Health of indigenous people in Africa. *The Lancet* **367**, 1937–1946.

Patin E, Laval G, Barreiro LB, Salas A, Semino O, *et al.* (2009). Inferring the demographic history of African farmers and Pygmy hunter–gatherers using a multilocus resequencing data set. *PLoS Genetics* 5: e1000448. doi: 10.1371/journal.pgen.1000448.

République du Congo. 2011 *Loi no. 2011-5 portant promotion et protection des droits des autochtones*. Brazzaville: Government of the Republic of Congo.

Verdu P. 2014 Population genetics of Central African Pygmies and non-Pygmies. In *Hunter-gatherers of the Congo Basin: Culture, history and biology of African Pygmies*. (ed Hewlett BS), pp. 31-58. New Brunswick (US) and London (UK): Transaction Publishers.

Verdu P., Verduemail, P., Austerlitz, F., Estoup, A. et al. (2009). Origins and genetic diversity of pygmy hunter-gatherers from western Central Africa. *Curr. Biol*. **19**, 312-318.

Wodon Q, Backiny-Yetna P, Ben-Achour A. 2012 Central Africa: The case of the Pygmies. In *Indigenous peoples, poverty and development*, (eds Hall G, Patrinos H) pp. 118-148, Cambridge: Cambridge University Press.
