# Supplementary material for: Distribution and Numbers of Pygmies in Central African Forests
Source: PLoS One. 2016 Jan 6;11(1):e0144499. doi: 10.1371/journal.pone.0144499 (PMC4711706; doi:10.1371/journal.pone.0144499)
Supplement: S2 Table — Source: Hoare AL. 2007 Resource rights and timber concessions: Integrating local peoples’ land-use practices in forest management in the Congo Basin. London: Rainforest Foundation-UK. (DOC) [file pone.0144499.s008.doc]

**S2 Table.** Empirical data of territory sizes for Pygmy camps in various localities in Central Africa (Source: Hoare AL. 2007 *Resource rights and timber concessions: Integrating local peoples’ land-use practices in forest management in the Congo Basin*. London: Rainforest Foundation-UK).

| **Pygmy group** | **Size of territory (km^2^)** | **Region / district** | **Observations** |
| --- | --- | --- | --- |
| Mbuti | 120-150 | Ituri (DRC) |  |
| Mbuti | 150-300 | Tetri region, Ituri (DRC) | Including overlapping areas |
| Mbuti | 160-170 | Tetri region, Ituri (DRC) | Not included overlapping areas |
| Aka | 400 | Lobaye (CAR) |  |
| Aka | 150 | Likouala (NE Congo) | Hunting areas |
| Aka | 70 | Likouala (NE Congo) | Gathering areas |
| Mbendjele | 4,831 | Berandjokou (N Congo) |  |
| Mbendjele | 5,964 | Linganga-Makao (Congo) |  |
| Mbendjele | 214 | Seke-Beye (Congo) |  |
| Mbendjele | 910 | Bangui-Motaba (Likouala, Congo) |  |
| Mbendjele | 2,239 | Mbandza (Congo) |  |
| Mbendjele | 3,970 | Minganga (Congo) |  |
| Mbendjele | 1,189 | Ngandzikolo (N Congo) |  |
| Mbendjele | 1,495 | Gatongo (Congo) |  |
| Mbendjele | 1,738 | Mobangi Bene & Mobangi Mboua (Congo) |  |
| Mbendjele | 872 | Toukoulaka Djello (Congo) |  |
| Mbendjele | 3,736 | Ibamba-Ikelemba (Congo) |  |
| Mbendjele & Nbombe Baka | 265 | Bomassa-Kabo (N Congo) |  |
| Not specified | 520 | E Cameroon | Hunting areas |
| Not specified | 81 | Mbomo (Congo) | Hunting areas |
| Not specified | 110 | Ikela (DRC) | Hunting areas |
| Not specified | 15 | Ikela (DRC) | Hunting areas |
| Not specified | 400 | NE Gabon | Hunting areas |
| Not specified | 211 | Kompia (Cameroon) | Hunting areas |
| Not specified | 160 | Kompia (Cameroon) | Hunting areas |
| Not specified | 355 | Kompia (Cameroon) | Hunting areas |
| Not specified | 300 | Kompia (Cameroon) | Hunting areas |
| Not specified | 452 | CAR | Hunting areas |
| Not specified | 130 | CAR | Hunting areas |
