# Supplementary material for: Distribution and Numbers of Pygmies in Central African Forests
Source: PLoS One. 2016 Jan 6;11(1):e0144499. doi: 10.1371/journal.pone.0144499 (PMC4711706; doi:10.1371/journal.pone.0144499)
Supplement: S3 Table — (DOC) [file pone.0144499.s009.doc]

**S3 Table. Predictor-variable sources** of the 34 predictor variables considered to build the environmental favourability model for Pygmies.

| Predictor variables | Source |
| --- | --- |
| Climate: |  |
| - *Maximum annual temperature* - *Minimum annual temperature* - *Maximum annual temperature range* - *Annual precipitation* - *Intra-annual pluviometric irregularity* (i.e. annual variation coefficient of monthly precipitation) | WorldClim (http://www.worldclim.org) |
| Topography: |  |
| - *Elevation* - *Slope* | GTOPO30 (US Geological Survey 1996) |
| Hydrography: |  |
| - *Distance to water masses* (i.e. lakes delimited as in http://www.naturalearthdata.com, and main river courses with water flow accumulation > 10^6^ cells, according to HydroSHEDS) - *Distance to minor rivers* (with water flow accumulation between 10^4^ and 10^6^ cells) | Hydrologically conditioned DEM of HydroSHEDS (http://hydrosheds.cr.usgs.gov) |
| Ecosystem types: |  |
| - *Broadleaf evergreen/semideciduous rainforests* (GC class 40) - *Swamp forests* (GC class 160) - *Deciduous forests* (GC class 50) - *Woody savannas* (GC classes 60 and 120) - *Shrublands* (GC classes 110 and 130) - *Grasslands* (GC class 140) - *Deserts* (GC class 200) | GlobCover (GC) Land Cover version 2.2 database for 2005-2006 (Bicheron et al. 2008) |
| - *Intact forest* | World Intact Forest Landscapes (IFL) Map in the year 2005 (Potapov et al. 2008) |
| Human concentration: |  |
| - *Rural population density* | LandScan™ 2008 High Resolution Global Population Data Set (copyrighted by UT-Battelle, LLC, operator of Oak Ridge National Laboratory), excluding any areas less than 2-km far from urban areas (as delimited by the MODIS 500-m Map of Global Urban Extent for 2001-2002 (Schneider et al. 2009; 2010) |
| - *Distance to populated places* | Administrative Centres & Populated Places shapefile at the Relational World Database II (RWDB2) updated in 2000 (http://www.fao.org/geonetwork) |
| Infrastructures: |  |
| - *Distance to roads* - *Distance to rail-roads* | Vector Map Level 0 at the Digital Chart of the World (DCW, http://worldmap.harvard.edu), updated in 2002 |
| Agriculture: |  |
| - *Intensive croplands* (GC class 14, including lands exclusively devoted to cropping) - *Cropland (>50%)/vegetation mosaics* (GC class 20) - *Vegetation (>50%)/cropland mosaics* (GC class 30) | GlobCover (GC) Land Cover version 2.2 database for 2005-2006 (Bicheron et al. 2008) |
| - *Non-intensive croplands* - *Global climate, soil and terrain slope constraints for cropping activities* | Food Insecurity, Poverty and Environment Global GIS Database (FGGD Digital Atlas for the year 2000) (FAO & IIASA 2007) |
| - *Percentage of area equipped for irrigation* | Global Map of Irrigation Areas (version 4.0.1) around the year 2000 (http://www.fao.org/nr/water) |
| Livestock: |  |
| - *Pasture and browse* | Food Insecurity, Poverty and Environment Global GIS Database (FGGD Digital Atlas for the year 2000) (FAO & IIASA 2007) |
| - *Density of poultry farms* - *Density of pigs* - *Density of cattle* - *Density of small ruminants (sheep, goats)* | FAO's Gridded Livestock of the World maps, derived from national data from 1992 to 2003 (Robinson *et al*. 2007) |
| Nature conservation policies: |  |
| - *Distance from protected areas* | World Database on Protected Areas (WDPA) (UNEP-WCMC 2012) |
| Exploitation of fauna: |  |
| - *Bushmeat extraction* | Fa et al. (2015) |

**References**

Bicheron, P. et al. (2008). *GlobCover 2005—Products description and validation report. Version 2.1. European Space Agency, Paris.* [http://ionia1.esrin.esa.int](http://ionia1.esrin.esa.int/).

Fa, J.E., Olivero, J., Real, R., Farfán, M.A., Márquez, A.L., Vargas, J.M., Ziegler, S., Wegmann, M., Brown, D., Margetts, B., Nasi, R. (2015). Disentangling the relative effects of bushmeat availability on human nutrition in central Africa. Scientific Reports 5:8168. DOI: 10.1038/srep08168.

FAO and IIASA (2007). *Mapping Biophysical Factors that Influence Agricultural Production and Rural Vulnerability* [van Velthuizen, H., Huddleston, B., Fischer, G., Salvatore, M., Ataman, E., Nachtergaele, F., Zanetti, M., Bloise, M.]. Environmental and Natural Resources Series No. 11. Rome.

Potapov, P. et al. (2008). Mapping the World's Intact Forest Landscapes by Remote Sensing. *Ecology and Society* 13:51-66.

Robinson, T.P., Franceschini, G., Wint, W. (2007). The Food and Agriculture Organization's Gridded Livestock of the World. *Veterinaria Italiana* 43:745-751.

Schneider, A., Friedl, M. A. & Potere, D. (2009). A new map of global urban extent from MODIS data. *Environ. Res. Lett.* 4:044003.

Schneider, A., Friedl, M. A. & Potere, D. (2010). Monitoring urban areas globally using MODIS 500m data: New methods and datasets based on urban ecoregions. *Remote Sens. Environ.* 114:1733-1746.

UNEP-WCMC (2012). *Data Standards for the World Database on Protected Areas*. UNEP-WCMC, Cambridge.

US Geological Survey (1996). *GTOPO30*. *Land processes distributed active archive center (LP DAAC), EROS data center.* https://lta.cr.usgs.gov/GTOPO30.
